# An oligo-His-tag of a targeting module does not influence its biodistribution and the retargeting capabilities of UniCAR T cells

**DOI:** 10.1038/s41598-019-47044-4

**Published:** 2019-07-22

**Authors:** Justyna Jureczek, Ralf Bergmann, Nicole Berndt, Stefanie Koristka, Alexandra Kegler, Edinson Puentes-Cala, Javier Andrés Soto, Claudia Arndt, Michael Bachmann, Anja Feldmann

**Affiliations:** 1 German Cancer Consortium (DKTK), partner site Dresden and German Cancer Research Center (DKFZ), Heidelberg, Germany; 2 University Cancer Center (UCC), Tumor Immunology, University Hospital Carl Gustav Carus, TU Dresden, Dresden, Germany; 3 Institute of Radiopharmaceutical Cancer Research, Helmholtz-Zentrum Dresden-Rossendorf (HZDR), Dresden, Germany; 4 Corporación para la Investigación de la Corrosión (CIC), Piedecuesta, Colombia; 5 Universidad de Santander, Cúcuta, Colombia; 6 National Center for Tumor Diseases (NCT), University Hospital Carl Gustav Carus, TU Dresden, Dresden, Germany

**Keywords:** Cancer immunotherapy, Cancer immunotherapy

## Abstract

Recently, we established the controllable modular UniCAR platform technology to advance the efficacy and safety of CAR T cell therapy. The UniCAR system is composed of (i) target modules (TMs) and (ii) UniCAR armed T cells. TMs are bispecific molecules that are able to bind to the tumor cell surface and simultaneously to UniCAR T cells. For interaction with UniCAR T cells, TMs contain a peptide epitope sequence which is recognised by UniCAR T cells. So far, a series of TMs against a variety of tumor targets including against the prostate stem cell antigen (PSCA) were constructed and functionally characterised. In order to facilitate their purification all these TMs are expressed as recombinant proteins equipped with an oligo-His-tag. The aim of the here presented manuscript was to learn whether or not the oligo-His-tag of the TM influences the UniCAR system. For this purpose, we constructed TMs against PSCA equipped with or lacking an oligo-His-tag. Both TMs were compared side by side including for functionality and biodistribution. According to our data, an oligo-His-tag of a UniCAR TM has only little if any effect on its binding affinity, *in vitro* and *in vivo* killing capability and *in vivo* biodistribution.

## Introduction

Chimeric antigen receptors (CARs) are synthetic receptors comprising an antigen recognition domain, mostly fused to the signalling domain of the CD3ζ chain with one or more co-stimulatory domain(s)^1–4^. CAR engineered immune cells can target surface antigens independently of MHC expression. While impressive clinical responses were reported in patients with hematological malignancies^5–15^, several obstacles prevent the broader application of the CAR technology especially for solid tumors. For instance, the expression of most if not all tumor-associated antigens (TAAs) is not limited to tumor cells. Varying levels of TAAs are also found on non-malignant cells of vital tissues. In order to increase the safety of the CAR technology and to minimise on-target/off-tumor toxicities but also other potentially life-threatening side effects, such as tumor lysis syndrome and cytokine release syndrome, a series of strategies have been developed including for example the use of suicide genes, the CRISPR/Cas9 system, targeting of co-expressed surface antigens, or gated targeting strategies^16–21^.

An alternative way to control the activity of artificial receptors is the imitation of natural ligand/receptor systems. Already in 2012, Urbanska *et al*. described such an artificial receptor system based on chicken avidin as artificial extracellular receptor domain instead of an anti-TAA antibody domain of a CAR^22^. T cells modified with such artificial avidin receptors are inactive but can interact with target cells via biotinylated adaptor molecules e.g. biotinylated antibodies^22,23^. However, the antigenicity of chicken avidin or bacterial streptavidin and the presence of natural anti-biotin antibodies in sera of healthy individuals^24^ might limit the use of such receptors in humans. To overcome such limitations we described in 2014 a modular CAR system termed UniCAR system^25^. Since then comparable switchable CAR strategies (e.g. sCARs) were described^26–28^. Like in conventional CARs the extracellular domain of UniCARs consists of an antibody domain. However, instead of a TAA, UniCARs recognise the human peptide sequence E5B9 (UniCAR epitope) that is part of the human nuclear autoantigen La/SS-B which is not present on living cells^29^. In contrast to the yeast peptide sequence used in sCARs^28^, the UniCAR epitope is proven even not to be immunogenic in patients suffering from autoimmune diseases and known to develop an immune response against the autoantigen La/SS-B. Moreover, even in case of an unexpected autoimmune response against La, anti-La autoantibodies have been described to be protective against anti-DNA autoantibodies. After adoptive transfer into patients UniCAR T cells remain inert until cross-linking to target cells. For cross-linkage of UniCAR T cells to a tumor cell, a bispecific adaptor molecule is required which we termed target module (TM). Consequently, the TM determines the specificity. Until now, we have proven *in vitro* and in experimental mice that UniCAR T cells can be retargeted to a broad spectrum of targets including for example to CD19, CD123, CD33, PSCA, PSMA, GD2, EGFR, and STn^30–36^.

The majority of UniCAR TMs are based on antibody domains. To facilitate their purification an oligo-His-tag (His-tag) is usually fused to the C-terminus. So far it is unclear whether or not the presence of this His-tag can affect the UniCAR system. Therefore, we decided to construct TMs with (His-tagged TM) or without (un-tagged) a His-tag and compared their functional and kinetic properties. For comparative analysis the well characterised prostate stem cell antigen (PSCA)-specific TM was used here, which can effectively redirect UniCAR T cells to tumor cells presenting PSCA^32^.

## Results

**Figure 1:**
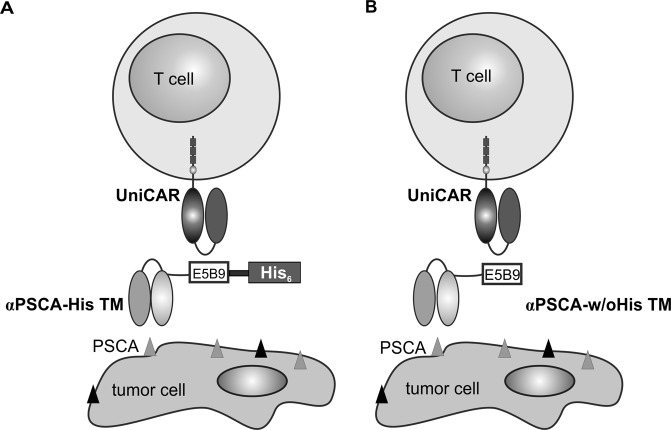
Schematic representation of the UniCAR system. The UniCAR system consists of two components: (i) T cells genetically modified with a universal chimeric antigen receptor (UniCAR) which is directed to the peptide epitope E5B9 (UniCAR epitope, E5B9) and (ii) a target module (TM). TMs are bifunctional molecules. Every TM contains the UniCAR epitope sequence E5B9. In addition to the UniCAR epitope a TM contains a binding domain which allows the TM to interact with a tumor-associated antigen on the cell surface of the target cell. Consequently, UniCAR T cells and TMs can form an immune complex. Moreover, the cross-linkage between UniCAR T cells and tumor cells leads to an activation of the UniCAR T cells and finally to the elimination of the tumor cells. UniCAR armed T cells are only switched on, when the TM is available, but automatically switched off when the TM is eliminated. Here we compare an αPSCA TM tagged with an oligo-His-tag ((**A**), αPSCA-His TM) with the same TM but lacking the His-tag ((**B**), αPSCA-w/oHis TM).

As summarised in the introduction section, the major aim of the presented work was to learn whether or not the presence of an oligo-His-tag in a UniCAR TM has an effect on the UniCAR system. The principle idea of the UniCAR system is schematically summarised in Fig. 1.

In order to enzymatically remove the oligo-His-tag, we (i) cloned a TM containing a recognition site for Tobacco Etch Virus (TEV) protease. The cleavage site was located upstream of the myc- and the oligo-His-tag but downstream of the UniCAR epitope E5B9. (ii) We established a cell line secreting this TM into cell culture supernatant. (iii) We isolated the TM from the cell culture supernatant via its His-tag using Nickel NTA affinity chromatography. (iv) After isolation we removed the His-tag via TEV protease cleavage and (v) separated the TM lacking the His-tag again via Nickel NTA affinity chromatography. (vi) We characterised the resulting un-tagged TM biochemically, (vii) analysed its functionality *in vitro* and *in vivo*, and finally (viii) compared its biochemical, functional properties as well as its biodistribution with the original His-tagged TM.

### Construction and production of the His-tagged and un-tagged TMs

**Figure 2:**
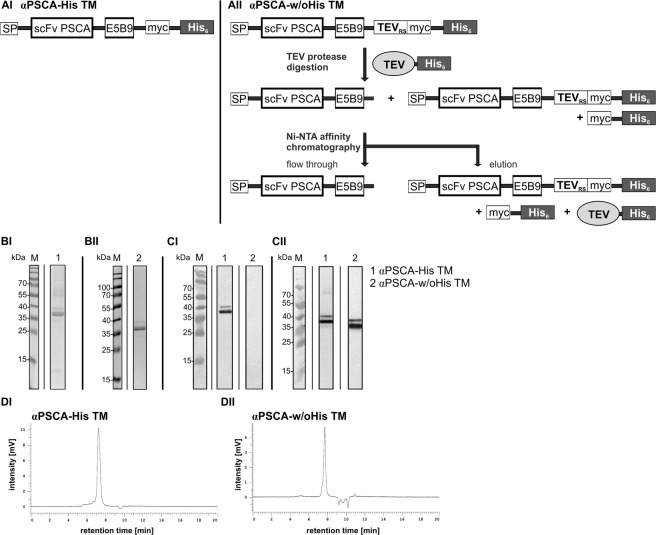
Design and purification of the αPSCA-His TM and the αPSCA-w/oHis TM. The previously described αPSCA-His TM that consists of the single-chain variable fragment (scFv) of the αPSCA mAb (MB1) and the E5B9 epitope for UniCAR recognition. (**AI**) In addition, the αPSCA-His TM is equipped with a myc- and a hexahistidine (His_6_)-tag for purification via Ni-NTA affinity chromatography. The secretion of the TM into cell culture supernatant is mediated by the N-terminal Ig kappa signal peptide sequence (SP). In contrast, the αPSCA-w/oHis TM was obtained in several steps. In order to remove the myc- and His-tag we introduced a TEV protease recognition site (TEV_RS_) N-terminally from the myc-tag and C-terminally from the scFv domain in the αPSCA-His TM. (**AII**) The resulting αPSCA-TEV_RS_-His TM was permanently produced by 3T3 cells and also purified via Ni-NTA affinity chromatography out of the supernatants. After TEV protease digestion of the purified TM material the remaining undigested αPSCA-TEV_RS_-His TM portion, the myc-His_6_-tag portion released from the αPSCA-TEV_RS_-His TM as well as the His-tagged TEV protease were separated from TEV-digested αPSCA-w/oHis TM by an additional Ni-NTA affinity chromatography step. After running a SDS-PAGE, the purified αPSCA-His TM (**BI**) and the TEV-digested, purified αPSCA-w/oHis TM (**BII**) fractions were stained with Coomassie brilliant blue G-250 (**BI**, **BII**) or blotted onto a nitrocellulose membrane for detection via αHis mAb (**CI**) or αE5B9 mAb (**CII**). M, molecular weight marker. Finally, the purity of purified αPSCA-His TM (**DI**) or TEV-digested, purified αPSCA-w/oHis TM (**DII**) were analysed by size exclusion HPLC (**DI**,**DII**).

We previously described the development and functionality of a PSCA-specific TM that is able to redirect UniCAR T cells to PSCA-positive tumor cells^32^. As schematically summarised in Fig. 2AI, this UniCAR TM was constructed by fusing variable heavy and light chain domains of the well described αPSCA monoclonal antibody (mAb) (clone MB1) to the UniCAR epitope^32,37–39^. For convenient purification from cell culture supernatant the TM was equipped with an oligo-His-tag at the C-terminus. For differentiation we renamed this TM here as αPSCA-His TM. Based on this construct we designed a novel TM which contained a TEV protease recognition site (TEV_RS_). The TEV_RS_ was introduced upstream of the myc- and His-tag but downstream of the UniCAR epitope E5B9 (Fig. 2AII). After transduction, TMs were permanently expressed in 3T3 cells as explained previously^32,35,36^ [see also METHODS]. After eukaryotic expression both (His-tagged) TMs were purified from cell culture supernatant using Nickel NTA affinity chromatography. The His-tag of the novel αPSCA-TEV_RS_-His TM was then enzymatically removed using TEV protease digestion followed by an additional Nickel NTA affinity chromatographic step. Thereby the His-tag released from the TM, remaining uncleaved TM and the His-tagged TEV protease were separated from the TM now lacking the His-tag (un-tagged TM). This un-tagged TM was termed αPSCA-w/oHis TM. As expected, the un-tagged TM was found in the flow through fraction.

Both αPSCA TMs, the isolated His-tagged (αPSCA-His TM) and the un-tagged (αPSCA-w/oHis TM) TM were biochemically analysed in parallel by SDS-PAGE (Fig. 2B) and immunoblotting (Fig. 2C). Both αPSCA TMs were well produced. Moreover, the UniCAR epitope E5B9 was accessible for αE5B9 mAb detection (Fig. 2CII). As also shown in Fig. 2C, we could clearly distinguish between the αPSCA-His TM (Fig. 2CI, lane 1) and the αPSCA-w/oHis TM (Fig. 2CI, lane 2). While the un-tagged TM is still reacting with the αE5B9 mAb recognising the UniCAR epitope (Fig. 2CII, lane 2), it is no more reacting with the mAb directed against the His-tag (Fig. 2CI, lane 2). These data indicate that the His-tag was successfully removed from the original protein.

The purity of the obtained TMs was confirmed by HPLC (Fig. 2D). The achieved purity of both TMs is comparable.

### Binding capability of the His-tagged and un-tagged TMs

**Figure 3:**
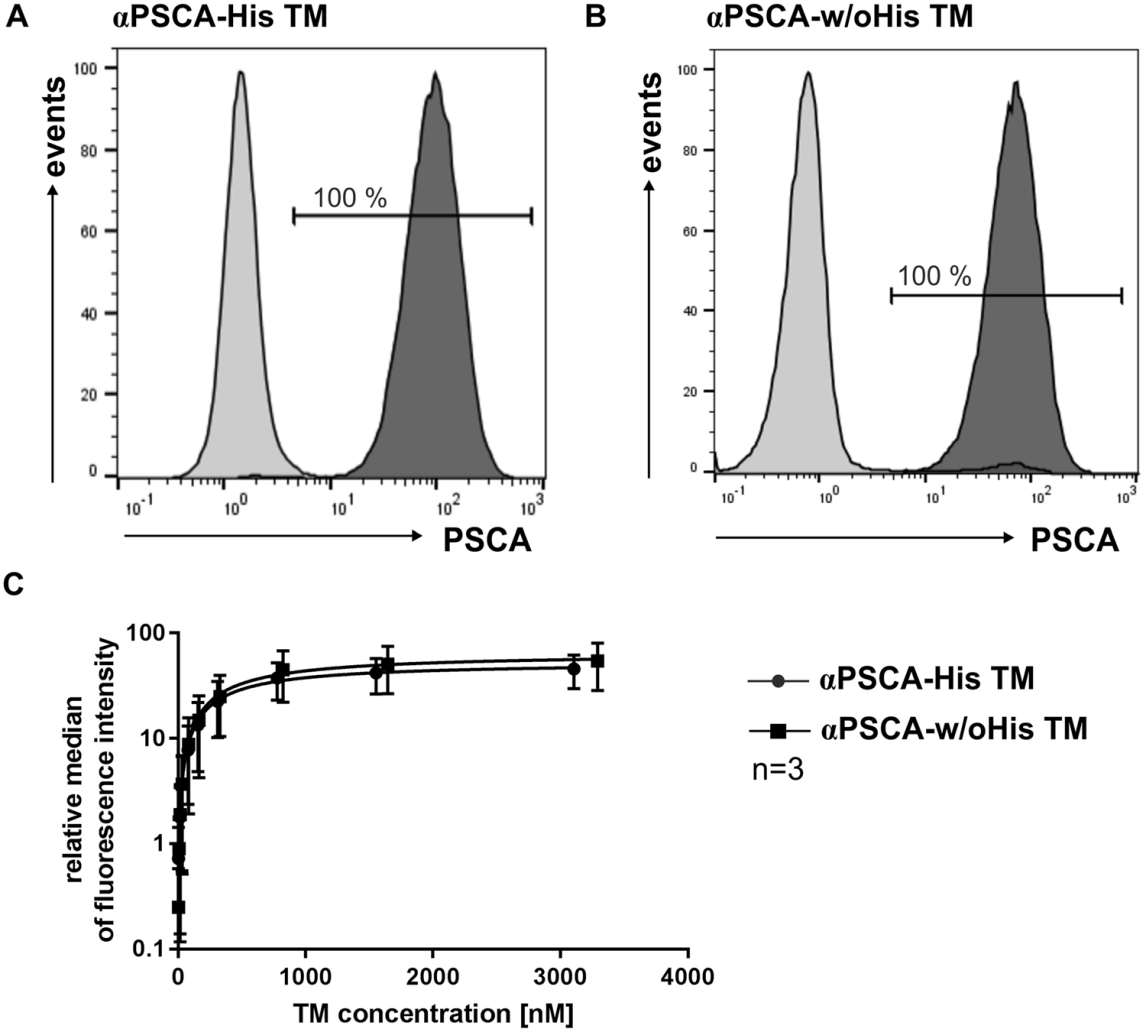
Binding analysis of the αPSCA-His TM and the αPSCA-w/oHis TM to PSCA expressing tumor cells. PC3-PSCA cells were incubated with αPSCA-His TM (**A**) or αPSCA-w/oHis TM (**B**). Either 25 ng/µl (**A**,**B**) or different concentrations of the respective TM (**C**) were used. TM binding was detected via the UniCAR epitope E5B9 using an αE5B9 mAb and a PE-conjugated α-mouse-IgG mAb. After staining, cells were measured by flow cytometry. PC3-PSCA cells that are positively stained with TMs are shown as dark grey graphs in the histograms (**A**,**B**). As negative control, the binding of the detection Abs in the absence of the TMs is shown in the bright grey graphs. Binding affinity curves were established for both TMs based on the mean and SEM of three independent experiments (**C**).

To compare the binding properties of the His-tagged and un-tagged TM, both TMs were incubated with PSCA expressing PC3 cells and their binding capability was estimated by flow cytometry analysis using an αE5B9 mAb recognising their C-terminal UniCAR epitope E5B9. As shown in Fig. 3(A–C), both TMs can efficiently bind to the target cells. Most importantly, after binding of both TMs to their target antigen PSCA, the UniCAR epitope of the TMs can still be recognised by the related αE5B9 mAb, which is an absolute prerequisite for the tumor-specific redirection of UniCAR T cells via TMs. In addition, increasing concentrations of both TMs were used to estimate binding affinities. As shown in Fig. 3C, we calculated comparable K_D_ values of 407 nM for the αPSCA-His TM and 476 nM for the αPSCA-w/oHis TM.

### Killing of PSCA expressing tumor cells by redirection of UniCAR T cells via His-tagged and un-tagged αPSCA TMs

In order to address the question, whether removal of the His-tag influences the functionality of the αPSCA scFv domain, chromium release assays were performed [see METHODS]. Human T cells were genetically modified to express signalling UniCARs comprising the co-stimulatory CD28 as well as the activating CD3ζ domain (UniCAR CD28/ζ) by lentiviral transduction as described previously^32^ [see also METHODS]. In addition, human T cells presenting UniCARs without any intracellular signalling domains (UniCAR stop) or expressing only the marker protein EGFP (vector control) were generated by lentiviral transduction and used as negative controls. For comparable reasons of different donors transduced T cells were sorted to >90% purity.

To analyse killing capabilities, UniCAR T cells were cultured together with ^51^Cr-loaded PC3-PSCA cells at an effector to target cell (E:T) ratio of 5:1 in the absence or presence of the respective TM for 24 h.

**Figure 4:**
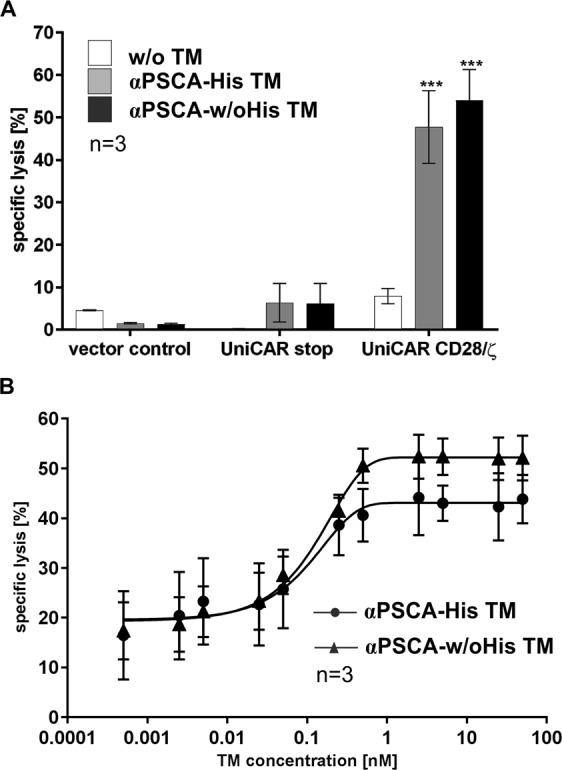
Comparison of specific tumor cell lysis by redirection of UniCAR T cells via the αPSCA-His TM and the αPSCA-w/oHis TM. In a standard chromium release assay UniCAR CD28/ζ armed T cells were co-cultivated with ^51^Cr-labelled PC3-PSCA tumor cells in the absence of TM (**A**, w/o TM) or in the presence of 50 nM (**A**) or indicated amounts of the respective TM (**B**) at an effector to target cell ratio of 5:1 for 24 h. Instead of UniCAR CD28/ζ armed T cells (**A**, UniCAR CD28/ζ) T cells expressing only EGFP marker protein (**A**, vector control) or the UniCAR stop construct missing any signalling domains (**A**, UniCAR stop) were used as controls. By using different TM amounts the range of working concentration was estimated (**B**). Mean of specific lysis and SEM are shown for three individual T cell donors. For statistical analysis two way ANOVA with Tukey’s (**A**) or Sidak’s (**B**) multiple comparisons test was performed. (***p < 0.001; with respect to vector control + TM, UniCAR stop + TM or UniCAR CD28/ζ w/o TM).

In agreement with previous data, only T cells armed with signalling UniCAR CD28/ζ constructs were able to effectively eradicate tumor cells in the presence of the His-tagged or un-tagged TM (Fig. 4A). In contrast, target cells were not attacked by modified T cells expressing UniCAR stop or only EGFP marker protein. As no specific lysis was detected in the absence of any TM (Fig. 4A, w/o TM), killing of PSCA-positive PC3 cell line occurred in a strictly TM-dependent manner. Both the His-tagged and the un-tagged TM can redirect UniCAR T cells to kill PSCA-positive target cells with similar efficacy. In order to calculate EC_50_ values of both TMs, titration experiments were performed (Fig. 4B). As expected, the killing correlates with the concentration of the respective TM. Increasing the concentration of both TMs (Fig. 4B) reaches a plateau at a concentration of about 1 nM. The calculated EC_50_ values for the αPSCA-His TM and the αPSCA-w/oHis TM were estimated at equal levels with 0.10 nM and 0.11 nM, respectively. Although the maximal tumor cell lysis mediated by the αPSCA-w/oHis TM in combination with UniCAR T cells seems to be slightly higher than using the αPSCA-His TM, there was no significant difference between the killing capacity of both TMs at any measured TM concentrations.

### Activation of UniCAR T cells redirected via His-tagged and un-tagged αPSCA TMs

**Figure 5:**
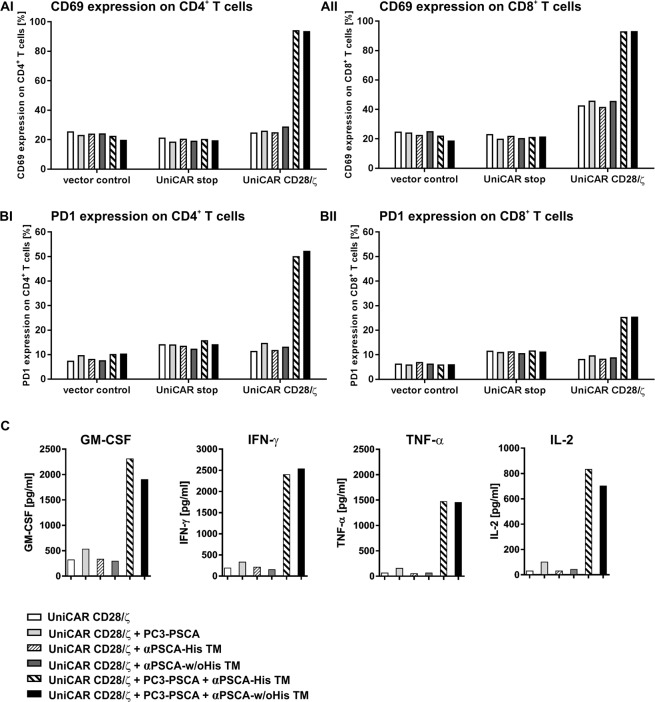
Activation and exhaustion of UniCAR T cells and cytokine release from UniCAR T cells redirected to PC3-PSCA tumor cells via the αPSCA-His TM and the αPSCA-w/oHis TM. UniCAR CD28/ζ expressing human T cells (UniCAR CD28/ζ) were incubated with or without PC3-PSCA tumor cells (effector to target cell ratio of 5:1) either in the absence or presence of 25 nM TM. All experiments were performed in triplicates. After 24 h, triplicates were pooled and CD69 (**AI**,**AII**) and PD1 (**BI**,**BII**) expression were estimated on CD4- (**AI**,**BI**) or CD8-positive (**AII**,**BII**) T cell subpopulations by staining with an αCD69-APC mAb or αPD1-PE mAb and flow cytometry. T cell subpopulations were discriminated by staining with an αCD4-PE-Vio770 and αCD8-VioBlue mAb. As additional controls, human T cells expressing only EGFP marker protein (vector control) or the UniCAR stop construct lacking any signalling domains (UniCAR stop) served. In addition, after 24 h of co-cultivation, supernatants of triplicates were pooled and afterwards cytokines secreted into cell culture supernatants were estimated using the MACSPlex Cytokine 12 kit (**C**). Shown cytokines were detected in relevant amounts. CD69, PD1 and cytokine profile for αPSCA-His TM and αPSCA-w/oHis TM are shown in comparison for one representative T cell donor.

In order to further verify the activation and exhaustion status of UniCAR CD28/ζ T cells, they were co-cultured with PSCA-positive target cells at an E:T ratio of 5:1 with or without 25 nM of the respective TM. As negative controls, T cells expressing UniCAR stop constructs or transduced with the vector control were used. After 24 h of co-cultivation, CD69 and PD1 surface expression on CD4- and CD8-positive T cell subpopulations were analysed by flow cytometry. As shown in Fig. 5A, an upregulation of CD69 activation marker occurs for both CD4^+^ (Fig. 5AI) and CD8^+^ (Fig. 5AII) UniCAR T cells in a strict tumor-specific and TM-dependent manner, while the activation level of UniCAR T cells redirected by the His-tagged or un-tagged TM was indistinguishable. In contrast, in the absence of target cells or TMs no CD69 upregulation was observed. Additionally, CD69 level was not increased using control T cells (vector control or UniCAR stop) in the presence of TM and target cells.

Similarly, the expression of PD1 surface marker on UniCAR CD28/ζ T cells is markedly increasing only in the presence of target cells and specific TM (Fig. 5B). The PD1 expression level was comparably high for His-tagged and un-tagged TM.

### Cytokine release from UniCAR T cells redirected via His-tagged and un-tagged αPSCA TMs

Amounts of cytokines secreted from redirected UniCAR T cells into cell culture supernatants were estimated using the MACSPlex Cytokine 12 kit (human) as described previously^31^ [see also METHODS]. For cytokine profile analysis, UniCAR CD28/ζ T cells were incubated alone or together with PSCA-positive PC3 target cells either with (25 nM) or without the respective TM for 24 h. Comparative cytokine profile for both αPSCA TMs containing or lacking the His-tag is shown for one representative donor in Fig. 5C. The secretion of the cytokines GM-CSF, IFN-γ, TNF-α, and IL-2 was clearly increased upon incubation of UniCAR CD28/ζ armed T cells together with PC3-PSCA target cells in the presence of respective αPSCA TM. No other cytokines, including IL-6, could be detected at relevant concentrations. In the negative controls (UniCAR CD28/ζ T cells either alone or with TM or with PC3-PSCA cells without TM) no or only background levels of cytokines could be measured. Consequently, collected data suggest that UniCAR T cells secrete pro-inflammatory cytokines in a strictly target-specific and TM-dependent manner. Obviously, the amounts of secreted cytokines were similar for both TMs containing or lacking the His-tag (Fig. 5C).

### Anti-tumor effects of UniCAR T cells redirected by His-tagged and un-tagged αPSCA TMs in experimental mice

**Figure 6:**
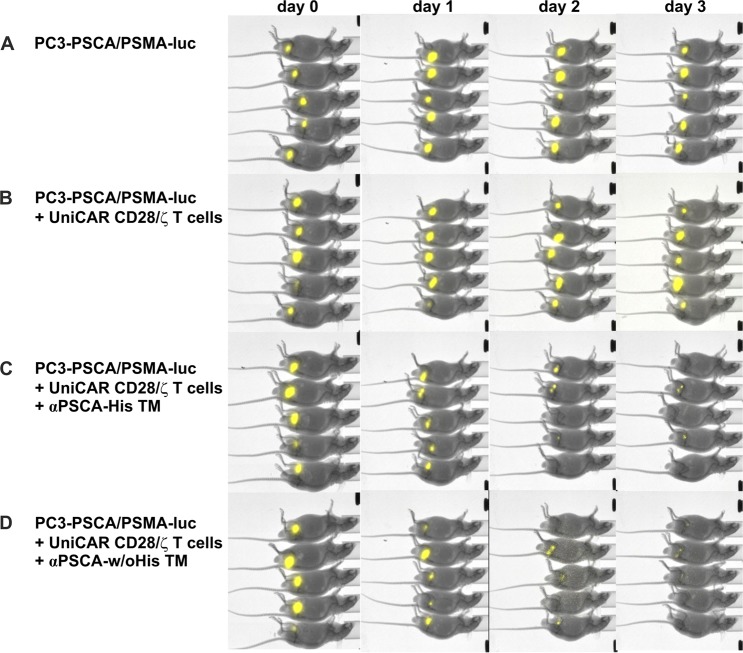
Tumor cell killing by redirection of UniCAR T cells via the αPSCA-His TM and the αPSCA-w/oHis TM *in vivo*. For studies in experimental mice four groups of male Rj:NMRI-Foxn1^nu/nu^ mice each consisting of five animals were distinguished. In the treated groups luciferase expressing PC3-PSCA/PSMA tumor cells (0.5 × 10^6^) were premixed with UniCAR CD28/ζ armed T cells (0.5 × 10^6^) in the presence of 10 µg αPSCA-His TM (**C**) or αPSCA-w/oHis TM (**D**) and subcutaneously co-injected into the right hind mouse flank. In the control mice only PC3-PSCA/PSMA-luc cells alone (**A**) or premixed together with UniCAR CD28/ζ armed T cells in the absence of any TM (**B**) were injected. Luminescence imaging of anesthetised mice was performed at day zero (day 0) and followed for three days (day 1, day 2, day 3).

To confirm the TM-dependent tumor killing capacity of UniCAR expressing T cells *in vivo*, a mouse tumor xenograft model was applied. Four groups of male Rj:NMRI-Foxn1^nu/nu^ mice each consisting of five animals were distinguished. Mixtures (100 µl) of 0.5 × 10^6^ firefly luciferase expressing PSCA/PSMA-positive PC3 cells (PC3-PSCA/PSMA-luc) together with 0.5 × 10^6^ UniCAR CD28/ζ T cells and 10 µg of His-tagged (Fig. 6C) or un-tagged (Fig. 6D) TM were subcutaneously injected in the right mouse flank. As controls served 0.5 × 10^6^ PC3-PSCA/PSMA-luc cells alone or mixed with 0.5 × 10^6^ UniCAR CD28/ζ T cells without any TM (Fig. 6A,B, respectively). Luciferase activity was analysed starting at day zero (Fig. 6, day 0), followed at day one (Fig. 6, day 1), day two (Fig. 6, day 2), and day three (Fig. 6, day 3). As shown in Fig. 6, in all treated animals no considerable luciferase activity was detectable already at day three, whereas in the control mice luciferase activity could easily be detected. According to these results UniCAR T cells can effectively kill tumor cells when armed with a TM. There was no obvious difference between the αPSCA-His TM and the αPSCA-w/oHis TM indicating that the His-tag has also no obvious effect on redirection of UniCAR T cells towards tumor cells *in vivo*.

### *In vivo* biodistribution of radiolabelled TMs

In order to visualise that TMs can bind at the tumor site *in vivo* and to compare the biodistribution and kinetics of the TMs in the Rj:NMRI-Foxn1^nu/nu^ mouse tumor model, the αPSCA-His TM and αPSCA-w/oHis TM were conjugated with NODAGA. According to MALDI-TOF analysis each TM was modified with approximately two NODAGA molecules. Afterwards the modified TMs were conjugated with ^64^Cu^2+^ showing a short positron range in order to get high-resolution PET images in experimental mice. The radiochemical purity reached 91 to 94% with specific activities from 28 to 40 GBq/µmol.

**Figure 7:**
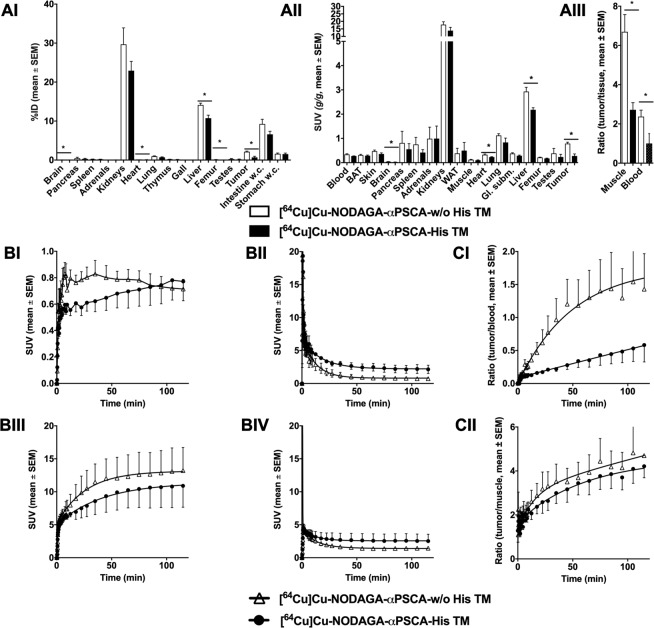
*In vivo* biodistribution and PET-biokinetics of ^64^Cu-radiolabelled TMs. (**A**) The biodistribution of the [^64^Cu]Cu-NODAGA-αPSCA-His TMs or [^64^Cu]Cu-NODAGA-αPSCA-w/oHis TMs was determined 120 min after single intravenous injection in male Rj:NMRI-Foxn1^nu/nu^ mice subcutaneously bearing luciferase expressing PC3-PSCA/PSMA tumors on the right hind leg by tissue and organ extraction. The distribution is presented as percentage of the total activity amount of the injected dose (**AI**, % ID) and the activity concentration in the tissues and organs (**AII**, SUV). In addition, the activity concentration is expressed as the ratios of tumor to muscle and tumor to blood (**AIII**). Mean and SEM of three animals are shown (**A**). For comparison of both TMs the student’s t-test was performed (*p < 0.05). (**B**,**C**) The kinetics of the [^64^Cu]Cu-NODAGA-αPSCA-His TMs or [^64^Cu]Cu-NODAGA-αPSCA-w/oHis TM were measured over 120 min after single intravenous injection in male Rj:NMRI-Foxn1^nu/nu^ mice bearing subcutaneous luciferase expressing PC3-PSCA/PSMA tumors on the right hind leg by dynamic small animal PET-imaging. The regions of interest (ROIs) were analysed for the tumors (**BI**), blood (**BII**), kidneys (**BIII**), liver (**BIV**), and the ratios of the tumor to blood (**CI**) and tumor to muscle (**CII**) were calculated. Mean and SEM of four animals are shown (**B**,**C**).

The results of the biodistribution experiments are summarised in Fig. 7A and the Tables 1 and 2. The biodistribution of the ^64^Cu-radiolabelled TMs was determined 120 min after single intravenous injection in male Rj:NMRI-Foxn1^nu/nu^ mice bearing subcutaneous luciferase expressing PC3-PSCA/PSMA tumors on the right hind leg by tissue and organ extraction. The activity amounts of both ^64^Cu-labelled TMs, the αPSCA-His TM as well as the αPSCA-w/oHis TM, were similar in the majority of analysed organs and tissues (Fig. 7AI–AII, Table 1, Table 2). However, the [^64^Cu]Cu-NODAGA-αPSCA-w/oHis TM accumulation (% ID) was significantly higher in the brain, heart, liver, femur, and tumor (Fig. 7AI, Table 1). Accordingly, the activity concentration (SUV) values of [^64^Cu]Cu-NODAGA-αPSCA-w/oHis TM were also higher in the tumor, brain, heart, and liver (Fig. 7AII, Table 2) and also for the tumor to background ratios (Fig. 7AIII, Table 2) in comparison to the [^64^Cu]Cu-NODAGA-αPSCA-His TM.

**Table 1:** Biodistribution of [^64^Cu]Cu-NODAGA-αPSCA-His TMs and [^64^Cu]Cu-NODAGA-αPSCA-w/oHis TMs in Rj:NMRI Foxn1^nu/nu^ PC3-PSCA/PSMA tumor bearing mice at 120 min after single intravenous injection.

organs	[^64^Cu]Cu-NODAGA-αPSCA-w/oHis TM % ID (n)	[^64^Cu]Cu-NODAGA-αPSCA-His TM % ID (n)	P
Brain	0.053 ± 0.006 (3)	0.040 ± 0.010 (3)	0.038
Pancreas	0.453 ± 0.465 (3)	0.287 ± 0.245 (3)	
Spleen	0.213 ± 0.119 (3)	0.187 ± 0.046 (3)	
Adrenals	0.037 ± 0.029 (3)	0.040 ± 0.044 (3)	
Kidneys	29.69 ± 7.305 (3)	22.94 ± 4.12 (3)	
Heart	0.193 ± 0.006 (3)	0.123 ± 0.015 (3)	0.001
Lung	0.980 ± 0.185 (3)	0.720 ± 0.193 (3)	
Thymus	0.200 ± 0.03 (3)	0.150 ± 0.036 (3)	
Gall	0.027 ± 0.006 (3)	0.020 ± 0.001 (3)	
Liver	14.13 ± 0.825 (3)	10.76 ± 1.31 (3)	0.019
Femur	0.133 ± 0.015 (3)	0.107 ± 0.006 (3)	0.037
Testes	0.267 ± 0.231 (3)	0.183 ± 0.162 (3)	
Tumor	2.13 ± 0.306 (3)	0.703 ± 0.493 (3)	0.013
Intestine w.c.	9.25 ± 2.110 (3)	6.61 ± 1.36 (3)	
Stomach w.c.	1.61 ± 0.350 (3)	1.54 ± 0.45 (3)	

The values show the mean and SD of the percentage of the total activity amount of the injected dose (% ID) in selected organs and the number of analysed animals (n). For comparison of both TMs the student’s t-test was performed. The calculated p values are shown if they are lower than 0.05. Intestine w.c., intestine with content; stomach w.c., stomach with content.

**Table 2:** Biodistribution of [^64^Cu]Cu-NODAGA-αPSCA-His TMs and [^64^Cu]Cu-NODAGA-αPSCA-w/oHis TMs in Rj:NMRI Foxn1^nu/nu^ PC3-PSCA/PSMA tumor bearing mice at 120 min after single intravenous injection.

Organs Tissues	[^64^Cu]Cu-NODAGA-αPSCA-w/oHis TM SUV (n)	[^64^Cu]Cu-NODAGA-αPSCA-His TM SUV (n)	P
Blood	0.339 ± 0.016 (3)	0.275 ± 0.011 (3)	
BAT	0.312 ± 0.046 (3)	0.289 ± 0.038 (3)	
Skin	0.474 ± 0.063 (3)	0.356 ± 0.065 (3)	
Brain	0.043 ± 0.005 (3)	0.026 ± 0.003 (3)	0.019
Pancreas	0.814 ± 0.676 (3)	0.555 ± 0.325 (3)	
Spleen	0.752 ± 0.41 (3)	0.418 ± 0.168 (3)	
Adrenals	0.988 ± 0.673 (3)	0.992 ± 0.732 (3)	
Kidneys	17.84 ± 2.640 (3)	13.85 ± 3.066 (3)	
WAT	0.383 ± 0.298 (3)	0.496 ± 0.477 (3)	
Muscle	0.121 ± 0.02 (3)	0.098 ± 0.024 (3)	
Heart	0.329 ± 0.041 (3)	0.231 ± 0.022 (3)	0.040
Lung	1.122 ± 0.1 (3)	0.834 ± 0.255 (3)	
Gl. subm.	0.376 ± 0.046 (3)	0.285 ± 0.039 (3)	
Liver	2.93 ± 0.249 (3)	2.18 ± 0.122 (3)	0.018
Femur	0.218 ± 0.014 (3)	0.174 ± 0.019 (3)	
Testes	0.383 ± 0.289 (3)	0.235 ± 0.155 (3)	
Tumor	0.787 ± 0.081 (3)	0.277 ± 0.100 (3)	0.007
Tumor / blood	2.371 ± 0.356 (3)	1.003 ± 0.408 (3)	0.018
Tumor / muscle	6.694 ± 1.242 (3)	2.721 ± 0.515 (3)	0.013

The values show the mean and SD of activity concentration in selected organs as SUV, the tumor to muscle and tumor to blood ratios and the number of analysed animals (n). For comparison of both TMs the student’s t-test was performed. The calculated p values are shown if they are lower than 0.05. Gl. subm., glandula submandibularis.

### *In vivo* PET-biokinetics of radiolabelled TMs

The dynamic PET measurements show similar behaviour of both radiolabelled TMs in the measured organs (Fig. 7BI–BIV) with a slight tendency of a faster blood clearance of the [^64^Cu]Cu-NODAGA-αPSCA-w/oHis TM (T_1/2_ 8.28 min) in comparison to [^64^Cu]Cu-NODAGA-αPSCA-His TM (T_1/2_ 12.5 min) resulting in a higher tumor accumulation for the [^64^Cu]Cu-NODAGA-αPSCA-w/oHis TM and improved tumor to blood ratio after 2 h (Fig. 7CI). The other tumor to background parameter, the tumor to muscle ratio, was similar for both TMs and reached values larger than four (Fig. 7CII).

### *In vivo* PET-imaging of radiolabelled TMs

**Figure 8:**
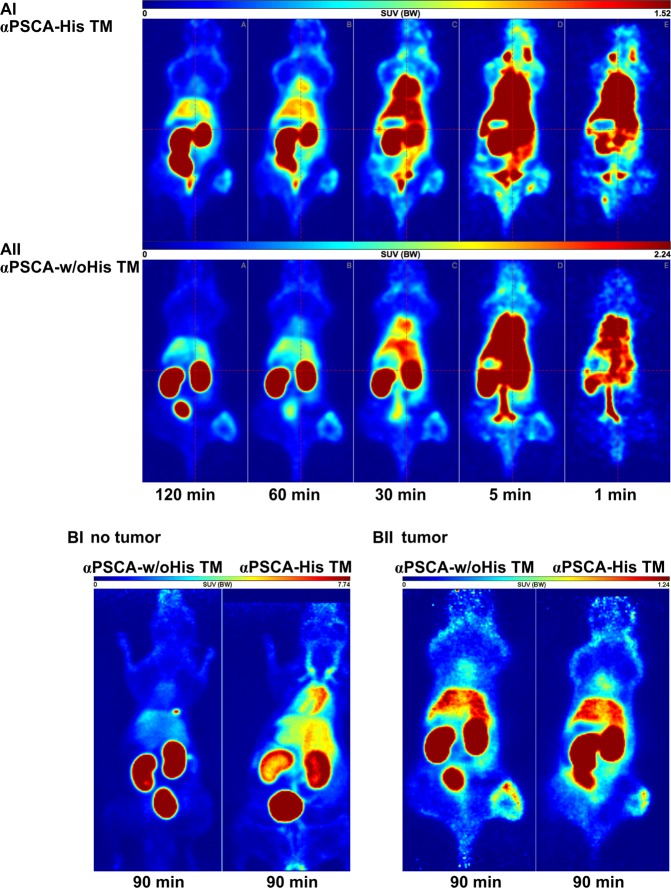
PET-images of ^64^Cu-radiolabelled TMs. [^64^Cu]Cu-NODAGA-αPSCA-His TMs or [^64^Cu]Cu-NODAGA-αPSCA-w/oHis TMs were intravenously injected in male Rj:NMRI-Foxn1^nu/nu^ mice with subcutaneous PC3-PSCA/PSMA-luciferase tumors. The images show the distribution of [^64^Cu]Cu-NODAGA-αPSCA-His TMs (AI) or [^64^Cu]Cu-NODAGA-αPSCA-w/oHis TMs (**AII**) as maximum intensity projections (MIPs) calculated for midframe times of 1, 5, 30, 60, and 120 min p.i. (**A**). In addition, the distribution of both radiotracers was imaged in tumor-free animals (**BI**) as well as in tumor bearing animals (**BII**) after 90 min p.i.

[^64^Cu]Cu-NODAGA-αPSCA-His TM or [^64^Cu]Cu-NODAGA-αPSCA-w/oHis TM were injected into Rj:NMRI-Foxn1^nu/nu^ mice bearing PC3-PSCA/PSMA-luciferase tumors. As shown in the PET-images (Fig. 8) biodistribution and kinetics of both the radiolabelled His-tagged (Fig. 8AI) and un-tagged (Fig. 8AII) TMs were similar in experimental mice 1, 5, 30, 60, 90, and 120 min p.i. Both the His-tagged as well as the un-tagged radiolabelled TMs allow the visualisation of established PC3-PSCA/PSMA-luciferase tumors and their heterogeneity in mice as the tumors were clearly separated from normal tissue (Fig. 8AI–II, 8BII). PET-images from tumor-free mice taken 90 min after injection of radiolabelled TMs demonstrate a lower blood pool activity of the [^64^Cu]Cu-NODAGA-αPSCA-w/oHis TM (Fig. 8BI).

## Discussion

CAR T cell therapy has shown impressive therapeutic effects in haematological malignancies^10–13^ which recently resulted in the approval of two of CD19-targeting CAR T cells by the U.S. FDA, tisagenlecleucel (Kymriah^®^– Novartis) in leukemia and lymphoma and axicabtagene ciloleucel (Yescarta^®^– Kite) in lymphoma. In spite of the impressing efficacy of these CAR T cell therapies still a lot of challenges remain to be solved especially before CAR T cells may also efficiently work for solid tumors. Among several problems of CAR T cell therapies, one big hurdle is that no target antigen is known strictly expressed only on tumor cells. As a consequence, CAR T cells are directed to TAAs being overexpressed on tumors. However, TAAs are not limited to tumor cells but the same targets may also be expressed on healthy organs and tissues, potentially leading to even fatal side effects. Moreover, the steering of CAR T cells is difficult. Once adoptively transferred into a patient, it is almost impossible to predict how the CAR T cells will behave in the patient. As a living drug, CAR T cells will proliferate once they find their target. Moreover, the proliferating activated CAR T cells will produce cytokines. However, at this time point only little is known about the tumor mass and how the individual CAR T cells will respond. Therefore, it is almost impossible to predict how CAR T cells will behave after adoptive transfer. As there is no safety switch in CAR T cells severe or even life-threating side effects such as tumor lysis syndrome and cytokine release syndrome may occur. In order to reduce the risk of such side effects, in 2014 we introduced the switchable modular UniCAR platform technology^25,30–36^. UniCAR T cells per se are inert. Their activity can be titrated via the application of a TM.

TMs are commonly bifunctional recombinant proteins. For a rapid purification, the TMs are equipped with an oligo-His-tag at the C-terminus. Although some recombinant His-tagged proteins are clinically used^40^, concerns about the presence of an oligo-His-tag in TMs of the UniCAR system came up during discussions with the legal authorities. Tagging of recombinant proteins with an oligo-His-tag is an ideal method for many applications because it allows an easy identification as well as rapid, high selective and efficient one-step purification via immobilized metal ion affinity chromatography (IMAC) of recombinant proteins. However, the His-tag and IMAC technologies may cause some problems. The His-tag may interfere with the biological activity of a protein^41^, has the potential for immunogenicity and can trap metal ions during IMAC^42^. Furthermore, it is known that the His-tag can influence the biodistribution of engineered scaffold proteins^43^. Because of safety reasons and regulatory requirements especially for the production of clinical-grade proteins substantial qualities are required including removing the His-tag. For His-tag removal from proteins after their purification, as an example TEV protease can be used showing a high stringency of sequence recognition, cleavage efficiency and suitability for large-scale protein preparation^44^.

We therefore wanted to learn whether or not removal of the His-tag is possible in principle and how the removal of the His-tag would affect the functionality and biodistribution of a UniCAR TM. To answer this question, we modified a previously described TM directed against the TAA PSCA^32,39^. For that purpose, we introduced a TEV protease cleavage site between the UniCAR epitope sequence and the His-tag which allowed us to enzymatically remove the oligo-His-tag. The isolated un-tagged TM was compared with the original His-tagged TM with respect to biochemical features, functionality and biodistribution. In principle we were able to successfully remove the His-tag from the C-terminus of the recently described αPSCA TM^32,39^ by TEV digestion without major effects on its functionality and biodistribution properties. As expected, TEV mediated His-tag removal from purified TMs is a more time-consuming and cost-intensive laboratory process than the rapid one-step purification of His-tagged TMs via Nickel NTA affinity chromatography. However, His-tag removal from the αPSCA TM by TEV digestion resulted in sufficient amounts of un-tagged TM with high purity. Obviously, in our study the His-tag at the C-terminus of the herein used αPSCA TM did not affect its therapeutic functionality in combination with the UniCAR system *in vitro* and *in vivo*.

Both the αPSCA-His TM and αPSCA-w/oHis TM redirect UniCAR T cells to kill PSCA expressing tumor cells equally well. Furthermore, the activation and exhaustion status as well as the cytokine profile of UniCAR T cells redirected by both TMs reach similar levels. In this context, marginal differences between both TMs can most probably be traced to donor heterogeneity. Interestingly, both CD4^+^ and CD8^+^ UniCAR armed T cell subpopulations get activated in a TM-dependent and tumor-specific manner. Whilst CD69 was equally upregulated on both T cell subpopulations, the PD1 expression level on CD8^+^ UniCAR T cells was clearly lower than on CD4^+^ UniCAR T cells. As we transduced total CD3^+^ T cells with UniCAR constructs, in this study we used a mixture of CD4^+^ and CD8^+^ UniCAR armed T cells showing a majority of CD8^+^ T cells (approximately 70%). However, we already published that not only CD8^+^ but also CD4^+^ UniCAR T cells, which were separately isolated, transduced and analysed, trigger a powerful antigen-specific tumor cell lysis with similar efficacy^45^. As shown in Supplementary Fig. S1(B), cytotoxicity of both UniCAR T cell subpopulations is mediated via the perforin/granzyme pathway. Furthermore, upon antigen- and TM-dependent stimulation, both UniCAR T cell subpopulations secrete pro-inflammatory cytokines GM-CSF, IFN-γ, TNF-α, and IL-2. CD4^+^ UniCAR T cells produce significantly more TNF-α and IL-2 than their CD8^+^ counterpart (see Supplementary Fig. S1(A)). According to these results, CD4^+^ and CD8^+^ UniCAR T cells possess effector functions within the UniCAR system in principle even separately from the other subset. A cytotoxic potential of CD8^+^ as well as CD4^+^ CAR armed T cell subsets against tumor cells was also shown by other authors^46,47^. However, they demonstrate differences in the effector functions of CD4^+^ and CD8^+^ CAR subsets and improved anti-tumor effects when combining both subsets^46,47^.

Regarding the comparison of His-tagged and un-tagged TMs with respect to pharmacokinetic properties and biodistribution *in vivo*, we observed a similar biodistribution pattern for both His-tagged and un-tagged αPSCA TM in experimental mice. Only in the minority of analysed organs or tissues like brain, heart, liver, femur, and PSCA expressing tumors the un-tagged TM was more accumulated than the His-tagged TM. Moreover, the un-tagged TM showed a slightly better tumor to blood ratio than the His-tagged TM. In contrast, other studies showed that His-tag based labelling of imaging probes increases the hepatic uptake of affibody molecules^48^ and renal reabsorption of nanobodies^49^. However, in agreement with other authors^43^, the influence of the His-tag depends on the composition of the engineered scaffold protein, the His-tag position within the molecule as well as on labelling and imaging procedures.

In summary, introducing a TEV protease cleavage site into the TM allowed us to remove the His-tag and isolate a His-tag-free TM. According to our data, removal of the His-tag had little effect if any on its binding affinity and killing capability both *in vitro* and *in vivo*. We only observed a slight yet not significant difference with respect to its biodistribution.

## Methods

### Cell lines

Murine 3T3, human HEK 293T, PC3-PSCA (recombinantly expressing PSCA), PC3-PSCA/PSMA (recombinantly co-expressing PSCA and PSMA), and PC3-PSCA/PSMA-luc (luciferase expressing) cell lines were gained, used and cultured as described previously^30–39^.

### Isolation, cultivation and lentiviral transduction of human T cells

Primary human T cells were isolated from healthy donors, genetically modified by lentiviral transduction, sorted, and cultured as described previously^30–36^. For transduction of human T cells lentiviral vectors were used encoding only the marker protein EGFP (vector control), the UniCAR stop construct without any signalling domains or the signalling UniCAR CD28/ζ construct containing co-stimulatory CD28 and activating CD3ζ domains. All the methods using human materials were performed in accordance with relevant local regulations and guidelines. All subjects gave their written informed consent. The local ethics committee of the medical faculty Carl Gustav Carus of the university hospital Carl Gustav Carus of the TU Dresden approved the study (EK27022006).

### Construction, expression and purification of TMs

Cloning, expression, purification, and functional analysis of the original His-tagged αPSCA-His TM was performed as described previously^32,39^.

In order to obtain the αPSCA-w/oHis TM a recognition site (ENLYFQ^G) for the TEV protease enzyme was inserted in the αPSCA-His TM. The protease cleavage site locates downstream of the αPSCA single-chain variable fragment (scFv) and the UniCAR epitope E5B9 sequences but upstream from the myc- and His-tag in the αPSCA-His TM construct. Thus, the resulting novel αPSCA-TEV_RS_-His TM construct consists of an Ig kappa signal peptide (SP), αPSCA scFv, E5B9, TEV recognition site (TEV_RS_), myc-tag, and His-tag. For cloning of the novel αPSCA-TEV_RS_-His TM the respective DNA fragment was ordered from Eurofins Genomics (Ebersberg, Germany). For permanent production of αPSCA-TEV_RS_-His TM, 3T3 cells were transduced with the respective lentiviral vector system and αPSCA-TEV_RS_-His TMs were purified by Ni-NTA affinity chromatography from cell culture supernatants. Cloning into the vector p6NST50, transduction of 3T3 cells and TM purification were performed as described previously^32–36^. After purification the imidazole containing samples were dialysed overnight against 1x PBS (Biochrom). Purity and concentration of the TMs were analysed by SDS-PAGE and immunoblotting^50–52^.

### Removal of the His-tag from the TM

In order to generate αPSCA-w/oHis TMs the eukaryotically produced and purified novel αPSCA-TEV_RS_-His TM was digested with TEV protease. TEV protease reaction was performed in 1x cleavage buffer (50 mM Tris-HCl pH 8.0, 0.5 mM EDTA, 2 mM DTT) at 4 °C for 24 h. Prior to the cleavage reaction the concentration of the purified and dialysed αPSCA-TEV_RS_-His TM fraction was adjusted to a concentration of 1 mg/ml in PBS. TEV protease (Sigma-Aldrich Chemie GmbH, München, Germany) was added at a protease to target protein ratio of 1:50 (w/w). After 24 h incubation time, the reaction mixture was passed again through a Ni-NTA column. The flow through fraction contained the αPSCA-w/oHis TM. Elution fractions contained uncleaved αPSCA-TEV_RS_-His TM, the His-tagged TEV protease and the cleaved oligo-His-tag. Isolated samples were dialysed and analysed by SDS-PAGE and immunoblotting.

### High-performance liquid chromatography

Size exclusion high-performance liquid chromatography (SE-HPLC) was performed to confirm the purity of the TM fractions as described previously^31,34^.

### Flow cytometry analysis

Specific binding of His-tagged and un-tagged TMs to PSCΑ-positive tumor cells was assessed by flow cytometry analysis. Briefly, 2 × 10^5^ of tumor cells were incubated with 25 ng/µl of the respective TM for 1 h. To estimate the binding affinity to PSCA and to determine the equilibrium dissociation constant (K_D_) values of the respective TM, target cells were incubated with increasing TM concentrations ranging between 0.1 ng/µl and 100 ng/µl. In order to detect specific TM binding, cells were subsequently stained with a mouse αE5B9 mAb directed against the UniCAR epitope E5B9 fused to the TM and finally with a PE-conjugated goat F(ab’)2 fragment α-mouse IgG-PE (Beckman Coulter GmbH, Krefeld, Germany) as detection Ab. MACSQuant® Analyzer and MACSQuantify® software (Miltenyi Biotec GmbH, Bergisch Gladbach, Germany) were used to analyse stained cells. Binding curves were created based on the relative median of fluorescence intensity of stained cells and K_D_ values were calculated with GraphPad Prism 7 software (GraphPad Software Inc., La Jolla, CA, USA).

### Cytotoxicity assay

Chromium release assays were performed to analyse whether redirected UniCAR CD28/ζ armed T cells can eliminate tumor cells. Therefore, ^51^Cr-labelled tumor cells were co-cultivated with UniCAR CD28/ζ armed T cells at an E:T ratio of 5:1 in the absence or presence of the respective TM. After 24 h of co-incubation, the specific tumor cell lysis was calculated as previously described^31–36^.

### T cell activation and cytokine release

For analysis of the activation and exhaustion status as well as the cytokine release, 5 × 10^4^ genetically modified T cells were incubated alone or together with 1 × 10^4^ target cells in the absence or presence of 25 nM of corresponding TM in 96-well plates in triplicates. After 24 h of co-cultivation, triplicates of cell-free supernatants were harvested and pooled to determine cytokine concentrations with the MACSPlex Cytokine 12 kit (Miltenyi Biotec GmbH) as described previously^31,32^. In parallel, triplicates of co-cultured T cells were pooled and were stained with αCD69-APC, αPD1-PE, αCD4-PE-Vio770, and αCD8-VioBlue mAbs (Miltenyi Biotec GmbH) and analysed by flow cytometry.

### Tumor xenograft model and optical imaging

For *in vivo* functionality test of both αPSCA TMs in combination with the UniCAR system we used a tumor xenograft model in immunodeficient mice as described previously^30–36^. Therefore five weeks old male Rj:NMRI-Foxn1^nu/nu^ mice (Janvier Labs, St. Berthevin, France) were divided in four groups each containing five animals. In all mice of the first control group only PC3-PSCA/PSMA-luc tumor cells expressing luciferase (0.5 × 10^6^) were subcutaneously injected into the right flank. Animals of the second group got tumor cells together with UniCAR CD28/ζ T cells (0.5 × 10^6^). Pre-mixtures of tumor cells, UniCAR CD28/ζ T cells and 10 µg of αPSCA-His TM or αPSCA-w/oHis TM were injected into animals of the treated groups. At day zero and the following three days, bioluminescence signals were acquired by optical imaging of anesthetised mice after injection of D-luciferin potassium salt. Optical imaging was performed as described previously^34^. All animal studies were approved by the Landesdirektion Dresden (24-9165.40-4. 24.9168.21-4/2004-1) and performed at the Helmholtz-Zentrum Dresden-Rossendorf (HZDR) in accordance with the guidelines of German Regulations for Animal Welfare.

### NODAGA conjugation and radiolabelling of the TMs

To visualise the *in vivo* binding of the respective TM at the tumor site and to compare the biodistribution and kinetics of both TMs in the Rj:NMRI-Foxn1^nu/nu^ mouse tumor model, the TMs were conjugated with NODAGA which is a chelator for radiometals like ^68^Ga^3+^ or ^64^Cu^2+^. As already published^31,33–35,53^ the NODAGA conjugation was performed in 0.1 M borate buffer pH 9.00 with approximately 20 nmol TM and with a surplus 40 times higher of p-SCN-NODAGA over 18 h at 25 °C. The TMs were purified by three times spin-filtration (Amicon Ultra-4, 10,000 MWCO) with 2 ml DPBS. The resulting proteins were analysed by MALDI-TOF that showed a mean value of two molecules NODAGA per molecule of TM. For radiolabelling the ^64^Cu^2+^ solution was adjusted to pH 5.5 with 2 mol/l NH_4_OAc and mixed with 1.6 nmol NODAGA-TM (50 µg) and incubated at 37 °C for 30 min. The samples were two times spin-filtrated (10,000 MWCO) with 2 ml DPBS containing 2 mM EDTA. The resulting radiochemical purity was analysed by ITL with the same buffer.

### *In vivo* biodistribution of radiolabelled TMs

Rj:NMRI Foxn1^nu/nu^ mice bearing a subcutaneous PC3-PSCA tumor (0.53 g ± 0.25 g) on the right hind leg and with a body weight of 26.3 g ± 1.3 g were intravenously injected with 0.5 MBq of ^64^Cu-labelled TMs. 120 min after injection, the activity of different collected tissues and organs were analysed. The activity amount was expressed as percentage of injected dose (% ID) and the activity concentration was calculated as standardised uptake values (SUV). The method was described in more detail previously^31,33–35,53,54^.

### *In vivo* small animal positron emission tomography (PET)

The procedures are described in detail elsewhere^31,33–35,53,54^. Radiolabelled TMs were intravenously injected in mice. To demonstrate the activity uptake, images were added with midframe times of 1, 5, 30, 60, 90, and 120 min and presented as maximum intensity projections (MIP). SUV were calculated and the kinetic data were generated as ^64^Cu-activity concentration time curves (mean ± SEM of four animals). The tumor to blood and tumor to muscle ratios were calculated as tumor to background (TBR) ratio.

### Statistical analysis

Statistical analysis was done as previously described^45^.

## Supplementary information

Dataset 1

## Data Availability

Data confirming the results of this study are presented in the manuscript and are available from the corresponding author upon reasonable request.
