# Supplementary material for: An oligo-His-tag of a targeting module does not influence its biodistribution and the retargeting capabilities of UniCAR T cells
Source: Sci Rep. 2019 Jul 22;9:10547. doi: 10.1038/s41598-019-47044-4 (PMC6646371; doi:10.1038/s41598-019-47044-4)
Supplement: Supplementary file 1 — Dataset 1 [file 41598_2019_47044_MOESM1_ESM.pdf]

Supplementary Information

An oligo-His-tag of a targeting module does not influence its biodistribution and the retargeting capabilities of UniCAR T cells

Justyna Jureczek, Ralf Bergmann, Nicole Berndt, Stefanie Koristka, Alexandra Kegler, Edinson Puentes-Cala, Javier Andrés Soto, Claudia Arndt, Michael Bachmann & Anja Feldmann

Supplementary Methods

Characterisation of $CD4^+$ or $CD8^+$ UniCAR T cells. Human $CD4^+$ or $CD8^+$ T cells were separately isolated, transduced to express UniCAR CD28/ζ constructs and used in activation assays as described previously[45]. In these activation assays UniCAR armed $CD4^+$ or $CD8^+$ T cells were cultured either alone or together with PC3-PSCA tumor cells in the absence or presence of αPSCA-His TM. After 24 h, cytokine secretion into cell culture supernatants was detected by ELISA. After 48 h, UniCAR CD28/ζ T cells were stained for intracellular granzyme B or perforin expression with mAbs directed against human granzyme B (REA226) and perforin (dG9) (Miltenyi Biotec GmbH) as described previously[45].

Supplementary Figure

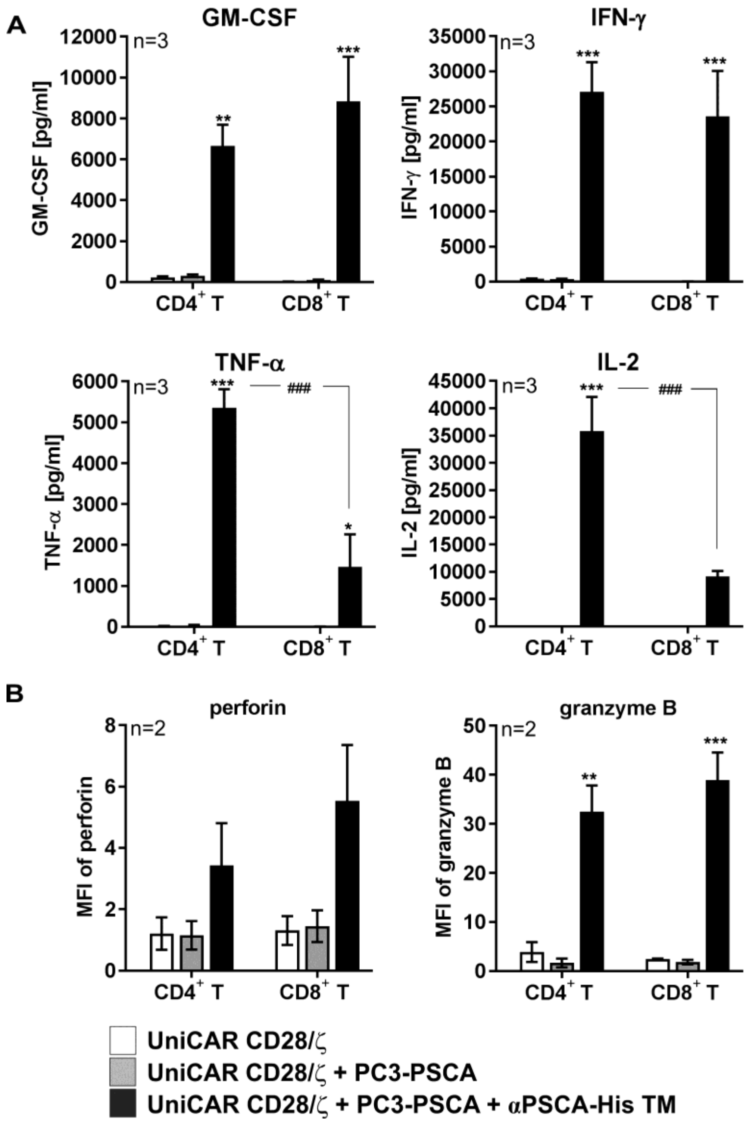

Supplementary Figure S1. Analysis of effector functions of redirected UniCAR armed CD4$^+$ or CD8$^+$ T cells. Human CD4$^+$ or CD8$^+$ T cells (T) were separately isolated and transduced with a lentiviral vector bicistronically expressing UniCAR CD28/ζ and EGFP marker protein. Subsequently, UniCAR CD28/ζ T cells (UniCAR CD28/ζ) were cultured alone or together with PC3-PSCA cells (effector to target cell ratio of 5:1) in the presence or absence of αPSCA-His TM. After 24 h, cytokine amounts were determined in the cell culture supernatants by ELISA (A). In addition, after 48 h, cells were stained for intracellular expression of granzyme B and perforin and the median fluorescence intensity (MFI) of these markers gated on CD4$^+$EGFP$^+$ or CD8$^+$EGFP$^+$ T cells was analysed by flow cytometry (B). Mean and SEM of three (A) or two (B) individual donors are shown. Statistical analysis was performed using the two way ANOVA with Tukey's and Sidak's multiple comparisons test. (*$p < 0.05$, **$p < 0.01$, ***$p < 0.001$, with respect to UniCAR CD28/ζ and UniCAR CD28/ζ + PC3-PSCA; $^{###}p < 0.001$, between CD4$^+$ and CD8$^+$ UniCAR CD28/ζ T cells).
